# Bioinformatic analysis of the molecular mechanisms underlying the progression of bone defects

**DOI:** 10.3389/fmed.2023.1157099

**Published:** 2023-06-08

**Authors:** Hao Liu, Xuan Zhao, Yin Li, Jiang Yi, Chenxi Zhang, Ziyang Zheng, Siming Dai, Guoyong Yin, Shujie Zhao

**Affiliations:** ^1^Department of Orthopedics, The First Affiliated Hospital of Nanjing Medical University, Nanjing, Jiangsu, China; ^2^Jiangsu Institute of Functional Reconstruction and Rehabilitation, Nanjing, Jiangsu, China; ^3^Spinal Cord Disease Research Center, Nanjing Medical University, Nanjing, Jiangsu, China

**Keywords:** bone defects, Kyoto encyclopedia of genes and genomes (KEGG), protein–protein interaction (PPI), circadian rhythms, metabolic pathway

## Abstract

**Background:**

The pathophysiology of bone defects (BDs) is complex, and the treatment for bone defects, in particular massive bone defects, remains a major clinical challenge. Our study was conducted to explore the molecular events related to the progression of bone defects a common clinical condition.

**Methods:**

First, microarray data of GSE20980 were obtained from the Gene Expression Omnibus (GEO) database, where 33 samples in total were used to analyze the molecular biological processes related to bone defects. Next, the original data were normalized and differentially expressed genes (DEGs) were identified. Additionally, Gene Ontology (GO) and Kyoto Encyclopedia of Genes and Genomes (KEGG) pathway enrichment analyses were conducted. Finally, a protein–protein interaction (PPI) network was constructed and the trends of the different genes were confirmed.

**Results:**

Compared with the samples of non-critical size defects (NCSD), the samples of critical size defects (CSD) had 2057, 827, and 1,024 DEGs at 7, 14, and 21 days post injury, respectively. At day 7, the DEGs were significantly enriched in metabolic pathways, at day 14 the DEGs were predominantly enriched in G-protein coupled signaling pathways and the Janus kinase (JAK)-signal transducer and activator of transcription (STAT) signaling pathway, and at day 21 the DEGs were mainly enriched in circadian entrainment and synaptic-related functions. The PPI network showed similar results. Quantitative real-time PCR (qRT-PCR) and western blot (WB) were performed to validate the partial results of sequencing.

**Conclusion:**

This study provides some clues about the molecular mechanism behind bone defects, which should contribute to scientific research and clinical treatment of this condition.

## Introduction

Bone defects often occur as a result of trauma, the resection of tumors, infections, osteoporosis, and other factors (1, 2). Millions of people worldwide suffer from bone defects every year, which can even cause severe disability (3). In the United States alone, there are more than 6.5 million patients with bone defects each year (4). Although there are many clinical methods to treat bone defects, such as autologous bone transplantation, no breakthrough has been made (5, 6). For this reason, it is imperative to understand the possible molecular mechanisms underlying the progression of bone defects in detail.

Physiologically, once the bone is damaged by mechanical injury, an inflammatory reaction is activated, and the repair cascade is initiated (7). Although the immune response and inflammatory-associated functions were found to play important roles (8), many of the key molecular changes that occur in a temporally specific manner remain unclear.

The transcriptome is known to reflect cellular pathophysiological information (9). In recent years, there have been few bioinformatic studies on bone defects. In this study, transcriptome data of GSE20980 were used to explore the molecular processes related to the progression of bone defects. We evaluated three different time points (7, 14, and 21 days post injury) for pathway and functional enrichment analyses. After that, a PPI network was constructed. We further validate the partial above results using quantitative real-time PCR (qRT-PCR) and western blot (WB). This study reveals the key molecular mechanisms behind the progression of bone defects and identifies potential therapeutic targets for the condition.

## Materials and methods

### Transcriptome data

The transcriptome data of GSE20980 based on the GPL1335 platform (Affymetrix Rat Genome 230 2.0 Array) were obtained from the National Center for Biotechnology Information Gene Expression Omnibus database.^1^ According to the traditional definition, a critical size defect (CSD) is the minimum defect size that cannot be healed spontaneously, where 8 mm is commonly considered as the CSD of rat calvarial defects (10–12). In this experiment, circular defects of 8 mm (CSD) or 4 mm (none-CSD, NCSD) were created in the calvaria by a drill. At the indicated time points post injury, the region of regeneration was harvested, and the RNA was isolated from the tissue using TRIzol reagent. Next, labeling and hybridization to rat whole-genome microarrays (Agilent) were performed.

### Data preprocessing

The raw data of the series were normalized using Robust Multichip Average (RMA) method and log2 transformed. Principal component analysis (PCA) was performed to visualize data variance. When different probes located the same gene symbol, we used the average to represent the gene expression level. In total, 31,042 gene chips were taken into consideration during the data processing.

### Identification and analysis of differentially expressed genes

We divided the data into six groups: CSD post-bone defects group vs. NCSD post-bone defects group at different times (7, 14, and 21 days). The Student’s *t*-test was used to identify DEGs with an average fold-change of >2.0, and *p* < 0.05 was considered to indicate a statistically significant difference.

### Pathway and functional enrichment analyses

Database for Annotation, Visualization, and Integrated Discovery (DAVID 6.8; http://david.abcc.ncifcrf.gov/) software was used to identify the enriched pathways and biological processes of the DEGs by Kyoto Encyclopedia of Genes and Genomes (KEGG) pathway and Gene Ontology (GO) functional analyses (GO terms were identified under categories of biological processes), respectively (13–15). A value of *p* < 0.05 was set as the threshold. The scatter plot was plotted by http://www.bioinformatics.com.cn, an online tool for data analysis and visualization.

### Protein–protein interaction network construction

The PPI network was constructed using the Search Tool for the Retrieval of Interacting Genes/Proteins (STRING; http://www.string-db.org/). The biological processes of the genes and proteins were visualized through the Cytoscape (version 3.7.1) software platform, using 400 as the default confidence cutoff (16, 17).

### Calvarial defect model

All animal experiments were approved by the Ethics Committee of Nanjing Medical University. 12 weeks old Sprague Dawley rats were used to performed calvarial defect models as previously described (12). In brief, under anesthetic conditions, defects (4 or 8 mm in diameter) were created on the right parietal bone of the skull using a round burr attached to drill. The defects were washed with saline.

### RNA isolation and qRT-PCR

Total RNA was extracted using the trizol reagent (Takara, Dalian, China), and the cDNA was amplified using the HiScript II QRT SuperMix for qPCR (R122-01, Vazyme, Nanjing, China). The qPCR was performed use a real-time 7500 PCR system (Applied Biosystems, Inc., United States) using AceQ qPCR SYBR Green Master Mix (Q111-02, Vazyme, China). All primer sequences are listed in Supplementary Table S1. The target genes were normalized to GAPDH expression, and the relative expression levels were performed using the 2^-△△CT^ method.

### Western blotting

Each bone callus was ground to fine particles with pestle and mortar in liquid nitrogen. Subsequently, the tissue was transferred to the EP tube for protein isolation. Equal amounts of proteins were separated via sodium dodecyl sulfate polyacrylamide gel electrophoresis and transferred to a polyvinylidene fluoride membrane. After blocked with 5% bovine serum albumin, the membrane was incubated overnight at 4°C with primary antibodies. The primary antibodies used were as follows: anti-phospho-JAK2 (1:1,000), anti-JAK2 (1:1,000), anti-phospho-Stat3 (1:1,000), anti-Stat3 (1:1,000), and anti-β-actin (1:1,000). Next, immunodetections were performed using the appropriate secondary antibodies (1:10,000), and the immunoreactive bands were visualized via the Tanon 4600SF System (Tanon, China).

### Statistical analysis

In all cases, data are presented as the means ± SEM from at least three independent biological replicates. GraphPad Prism 9.0.0 (GraphPad Software, La Jolla, CA, United States) and SPSS software version 26.0 (SPSS, Inc., Chicago, IL, United States) were used to conduct statistical analyses. Unpaired two-tailed Student’s *t*-test was used for comparisons between two groups. Differences between groups were considered significant at a value of *p* < 0.05.

## Results

### Data preprocessing and differentially expressed gene screening

**Figure 1:**
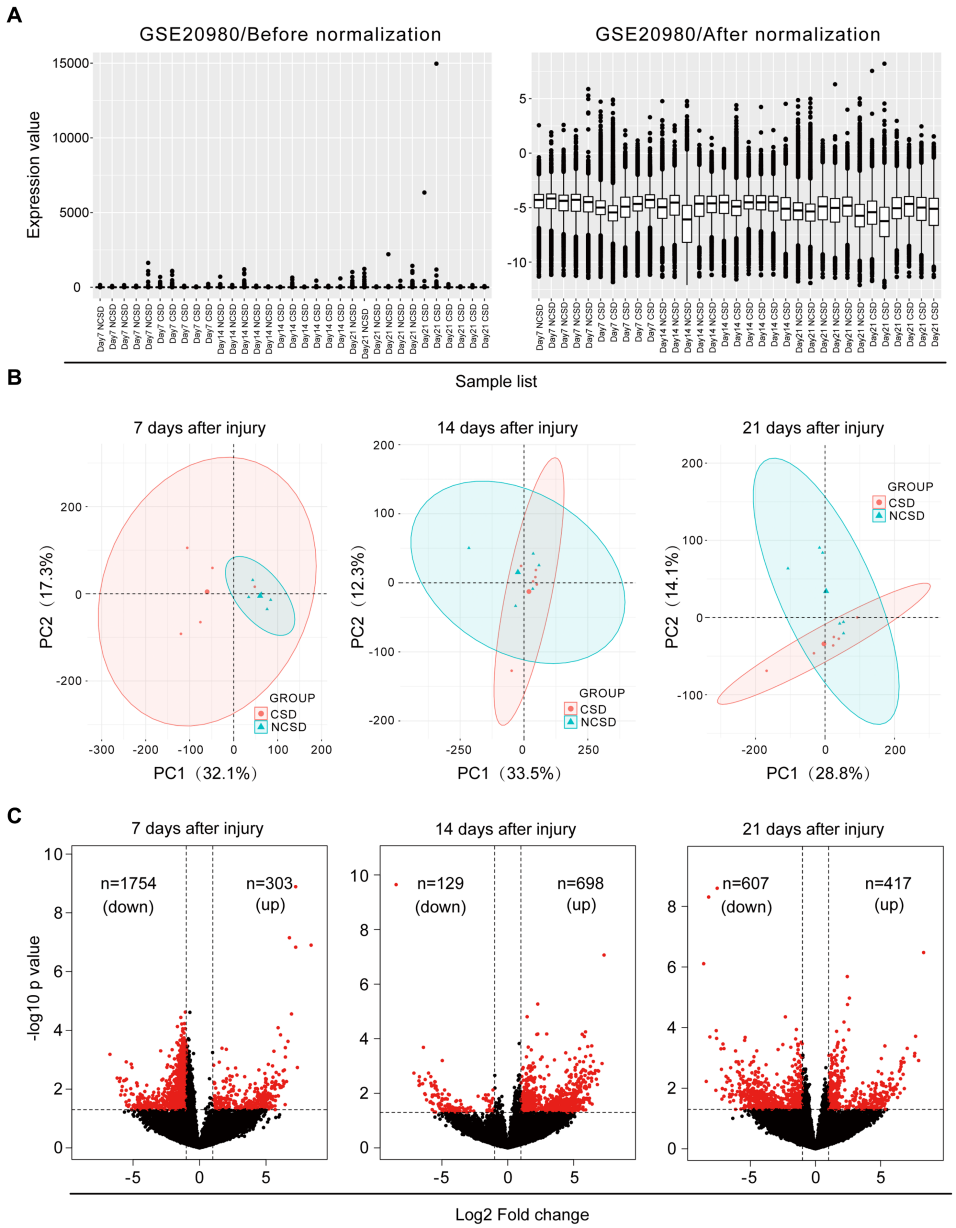
**(A)** Data normalization of differentially expressed genes (DEGs). Box plots of gene expression in the critical size defect (CSD) and non-critical size defect (NCSD) at the new bone site (left panel) before and (right panel) after normalization. **(B)** Principal component analysis and **(C)** Volcano plots at 1–3  weeks of the CSD vs. NCSD groups.

**Figure 2:**
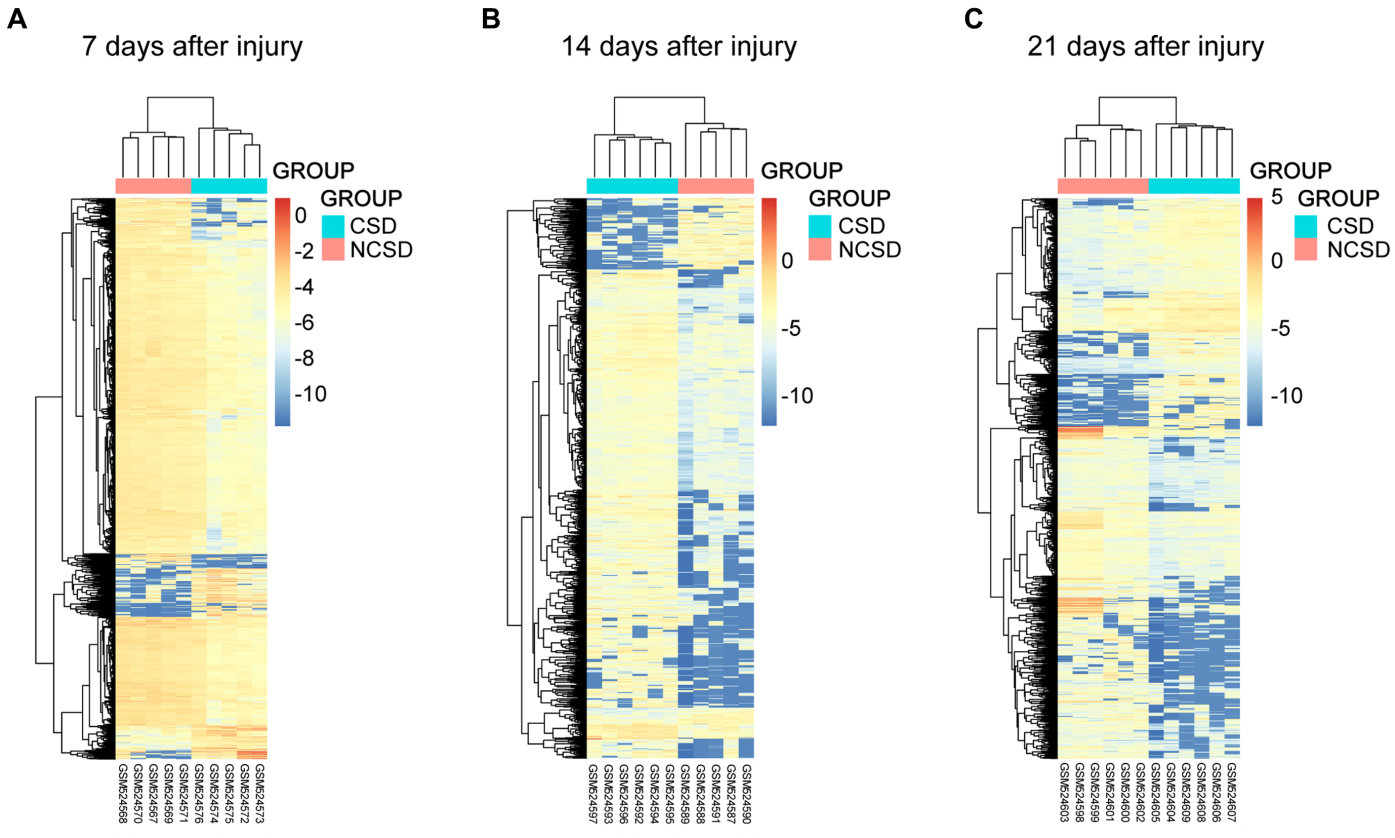
Heat maps of the genes at 1 **(A)**, 2 **(B)**, and 3 **(C)** weeks after bone defect in the CSD vs. NCSD groups. Horizontal axis represents each sample, and the vertical axis represents each gene. Blue and red colors represent low and high expression values, respectively.

Box plots of the CSD and NCSD data at different time points (7, 14, and 21 days) before and after normalization are presented in Figure 1A. The results demonstrated that after normalization, the expression values of each sample were similar. Principal component analysis (PCA) captures the variance of the principal components, and our study shows that overall gene expression is different across the CSD and NCSD groups at the three time points (Figure 1B). The DEGs between the CSD groups and NCSD groups at the three time points were analyzed following data preprocessing and the PCA. As shown in Figure 1C, there were 303, 698, and 417 upregulated DEGs at days 7, 14, and 21, respectively. Additionally, 1,754, 129, and 607 downregulated DEGs were identified at the three time points, respectively. At day 14, the number of upregulated DEGs was more than the downregulated ones. In contrast, at days 7 and 21, there were much less upregulated DEGs. The volcano plots that were constructed to visualize these identified DEGs are shown in Figure 1C. The heat maps of color-coded gene expression values, indicating the variability in DEGs expression between the CSD and NCSD groups after the bone injury, are shown in Figures 2A–C.

### KEGG pathway and GO enrichment analyses

**Figure 3:**
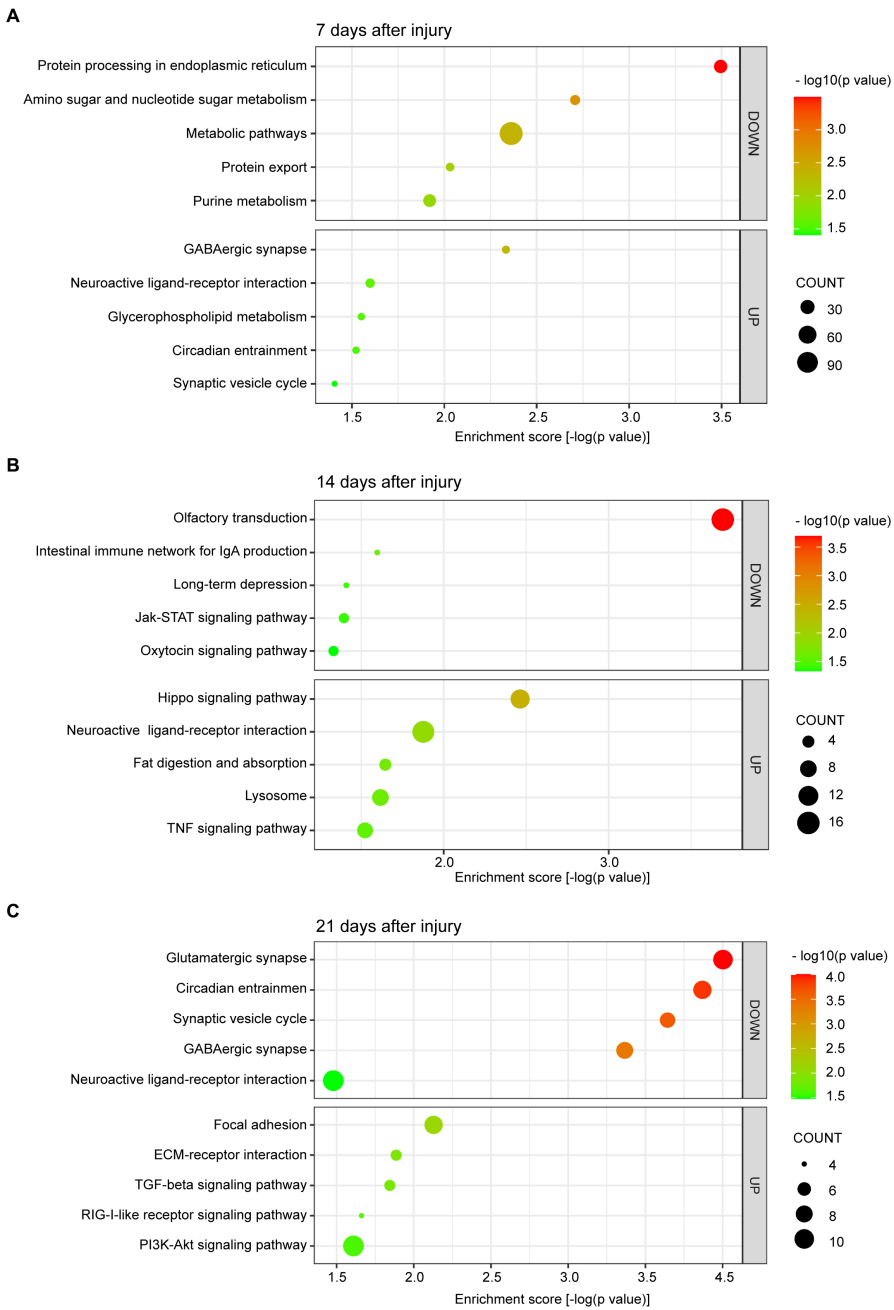
Scatter plot of enriched KEGG pathways for DEGs at 7 **(A)**, 14 **(B)**, and 21 **(C)** days after bone defect. The color and size of the dots represent the range of −log10 (*p* value) and the number of DEGs mapped to the indicated pathways, respectively. KEGG, Kyoto Encyclopedia of Genes and Genomes. DEGs, differentially expressed genes.

In this study, we concentrated on the DEGs at 7, 14, and 21 days post injury. The most enriched KEGG pathways of the up- and downregulated DEGs at 7, 14, and 21 days are shown in Figures 3A–C, respectively. At day 7, the upregulated DEGs were primarily associated with synaptic-related functions, including the GABAergic synapse (*p =* 4.64 × 10^−03^), neuroactive ligand-receptor interaction (*p =* 2.52 × 10^−02^), and synaptic vesicle cycle (*p =* 3.92 × 10^−02^; Figure 3A). At day 14, the upregulated DEGs were highly associated with the Hippo signaling pathway (*p =* 3.44 × 10^−03^), neuroactive ligand-receptor interaction (*p =* 1.33 × 10^−02^), fat digestion, and absorption (*p =* 2.26 × 10^−02^; Figure 3B). At day 21 post injury, the upregulated DEGs were enriched in focal adhesion (*p =* 7.44 × 10^−03^), ECM-receptor interaction (*p =* 1.30 × 10^−02^), the TGF-β signaling pathway (*p =* 1.43 × 10^−02^), the RIG-I-like receptor signaling pathway (*p =* 2.18 × 10^−02^), and the PI3K-Akt signaling pathway (*p =* 2.46 × 10^−02^; Figure 3C).

We are more concerned about the downregulated DEGs. Additionally, at day 7, the downregulated DEGs were involved in metabolic pathways, including protein processing in endoplasmic reticulum (*p =* 3.20 × 10^−04^), amino sugar and nucleotide sugar metabolism (*p =* 1.96 × 10^−03^), metabolic pathways (*p =* 4.35 × 10^−03^), protein export (*p =* 9.31 × 10^−03^), and purine metabolism (*p =* 1.20 × 10^−02^; Figure 3A). At day 14, the downregulated DEGs were primarily enriched in pathways related to olfactory transduction (*p =* 2.03 × 10^−04^), intestinal immune network for IgA production (*p =* 2.53 × 10^−02^), long-term depression (*p =* 3.90 × 10^−02^), the Janus kinase-signal transducer and activator of transcription (JAK–STAT) signaling pathway (*p =* 4.03 × 10^−02^), and the oxytocin signaling pathway (*p =* 4.66 × 10^−02^; Figure 3B). Finally, at day 21 post injury, the downregulated DEGs were enriched in pathways related to synaptic-related functions and circadian entrainment, including the glutamatergic synapse (*p =* 9.93 × 10^−05^), circadian entrainment (*p =* 1.35 × 10^−04^), synaptic vesicle cycle (*p =* 2.27 × 10^−04^), GABAergic synapse (*p =* 4.30 × 10^−04^), and neuroactive ligand-receptor interaction (*p =* 3.32 × 10^−02^; Figure 3C).

The top 5 GO terms (biological processes) of the up- and downregulated DEGs are all summarized in Table 1. The results showed that at 7 days post injury, the upregulated DEGs were mostly enriched in the single-multicellular organism process (*p =* 3.41 × 10^−13^), system process (*p =* 4.07 × 10^−13^), and multicellular organismal process (*p* = 4.97 × 10^−13^). At other two time points, the upregulated DEGs were enriched in some development and cellular processes, which contained the multicellular organismal process (*p =* 5.18 × 10^−12^), single-multicellular organism process (*p =* 1.10 × 10^−11^), single-organism cellular process (*p =* 1.28 × 10^−11^), anatomical structure development (*p =* 1.10 × 10^−09^), and system development (*p =* 2.20 × 10^−09^) terms at 14 days, and multicellular organismal process (*p =* 1.79 × 10^−16^), single-multicellular organism process (*p =* 2.78 × 10^−15^), response to chemical (*p =* 2.45 × 10^−12^), reproduction (*p =* 6.58 × 10^−08^), and system process (*p =* 9.46 × 10^−08^) terms at 21 days.

**Table 1:** GO terms enriched by differentially expressed genes at three time-points following bone defect.

A, CSD vs. NCSD (day 7)				
Category	GO ID	Biological process	*p* v*alue*	Rank
Upregulated	GO:0044707	Single-multicellular organism process	3.41e-13	1
	GO:0003008	System process	4.07e-13	2
	GO:0032501	Multicellular organismal process	4.97e-13	3
	GO:0050877	Neurological system process	2.83e-12	4
	GO:0098916	Anterograde trans-synaptic signaling	2.14e-11	5
Downregulated	GO:0044237	Cellular metabolic process	8.87e-20	1
	GO:0006396	RNA processing	3.00e-19	2
	GO:0071704	Organic substance metabolic process	2.42e-18	3
	GO:0008152	Metabolic process	1.20e-17	4
	GO:0016071	mRNA metabolic process	3.02e-17	5
B, CSD vs. NCSD (day 14)				
Category	GO ID	Biological process	*p value*	Rank
Upregulated	GO:0032501	Multicellular organismal process	5.18E-12	1
	GO:0044707	Single-multicellular organism process	1.10E-11	2
	GO:0044763	Single-organism cellular process	1.28E-11	3
	GO:0048856	Anatomical structure development	1.10E-09	4
	GO:0048731	System development	2.20E-09	5
Downregulated	GO:0003008	System process	3.52E-10	1
	GO:0007186	G-protein coupled receptor signaling pathway	1.95E-09	2
	GO:0050877	Neurological system process	4.97E-09	3
	GO:0050906	Detection of stimulus involved in sensory perception	1.47E-08	4
	GO:0007600	Sensory perception	4.15E-08	5
C, CSD vs. NCSD (day 21)				
Category	GO ID	Biological process	*p* value	Rank
Upregulated	GO:0032501	Multicellular organismal process	1.79E-16	1
	GO:0044707	Single-multicellular organism process	2.78E-15	2
	GO:0042221	Response to chemical	2.45E-12	3
	GO:0000003	Reproduction	6.58E-08	4
	GO:0003008	System process	9.46E-08	5
Downregulated	GO:0098916	Anterograde trans-synaptic signaling	1.02E-09	1
	GO:0099537	Trans-synaptic signaling	1.06E-09	2
	GO:0099536	Synaptic signaling	1.49E-09	3
	GO:0007267	Cell–cell signaling	1.51E-08	4
	GO:0007268	Chemical synaptic transmission	2.60E-08	5

GO, gene ontology.

At 7 days post injury, the downregulated DEGs were mostly enriched in the metabolic process, such as cellular metabolic process (*p =* 8.87 × 10^−20^), organic substance metabolic process (*p =* 2.42 × 10^−18^), metabolic process (*p =* 1.20 × 10^−17^), and mRNA metabolic process (*p =* 3.02 × 10^−17^) terms. At 14 days post injury, they were remarkably associated with the system process (*p =* 3.52 × 10^−10^), G-protein coupled receptor signaling pathway (*p =* 1.95 × 10^−09^), and neurological system process (*p =* 4.97 × 10^−09^) terms. Finally, downregulated DEGs were enriched in neurological and synaptic-related functions at 21 days post injury, which were anterograde trans-synaptic signaling (*p =* 1.02 × 10^−09^), trans-synaptic signaling (*p =* 1.06 × 10^−09^), synaptic signaling (*p =* 1.49 × 10^−09^), cell–cell signaling (*p =* 1.51 × 10^−08^), and chemical synaptic transmission (*p =* 2.60 × 10^−08^).

### PPI network construction and functional module analysis

**Figure 4:**
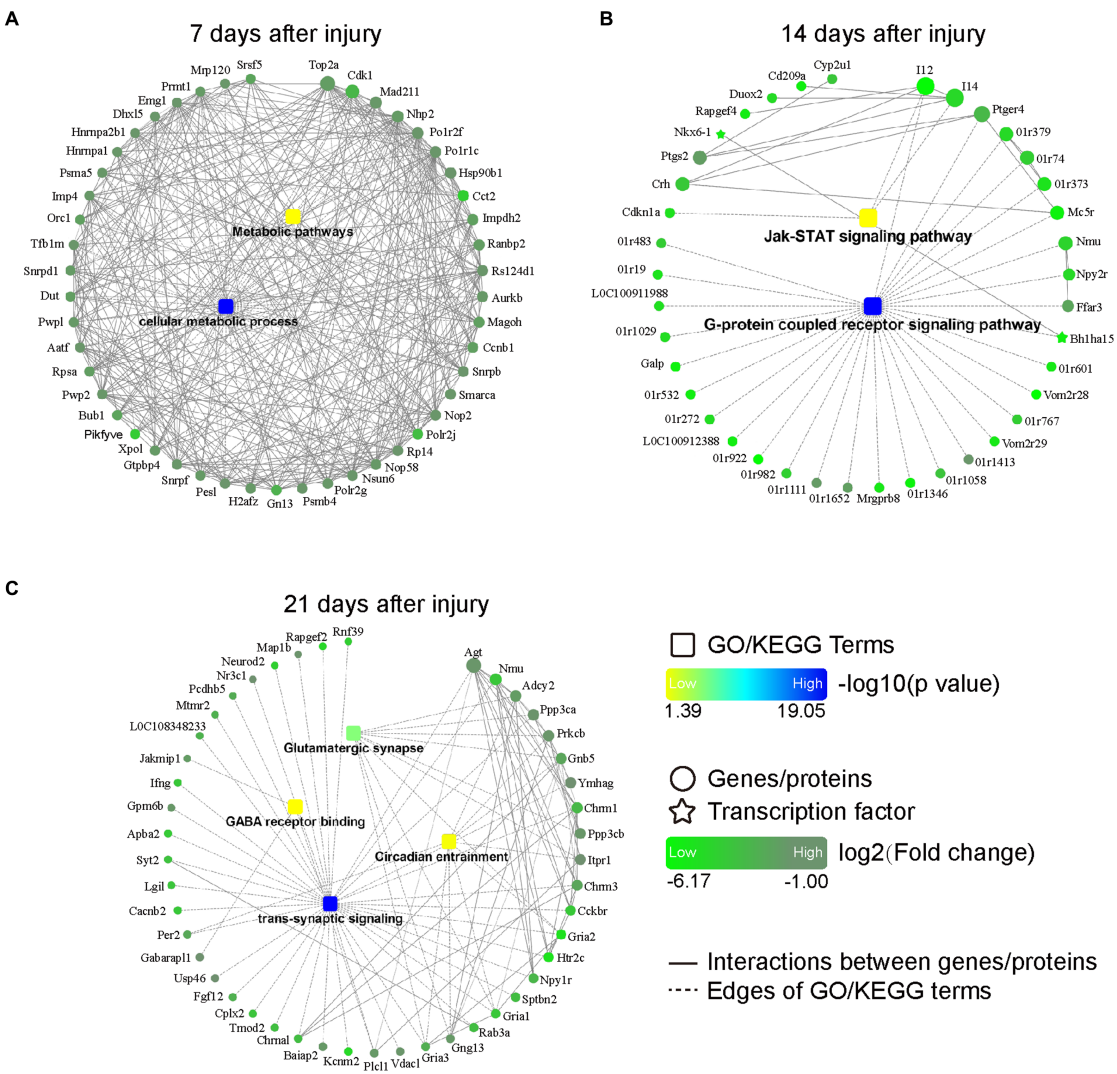
PPI networks based on DEGs at 7 **(A)**, 14 **(B)**, and 21 **(C)** days after bone defect. Rectangular nodes indicate a biological process or a KEGG pathway, colored with gradient colors from yellow (smaller value of *p*) to blue (larger value of *p*). Circular nodes indicate genes/proteins. Star-shaped nodes indicate transcription factor. Light green-to-dark green colors indicate low-to-high log2 (fold change). Interactions are shown as solid lines between genes/proteins, and edges of KEGG pathways/Go terms are presented as dashed lines. DEGs, differentially expressed genes; PPI, protein–protein interaction; KEGG, Kyoto Encyclopedia of Genes and Genomes; GO, Gene Ontology.

The PPI networks of DEGs were constructed via STRING and visualized by Cytoscape software. The results revealed that the downregulated DEGs were enriched in the metabolic pathways and cellular metabolic processes at 7 days post injury (Figure 4A); in the JAK–STAT signaling pathway and G-protein coupled receptor signaling pathway at 14 days post injury (Figure 4B); and in the glutamatergic synapse, GABA receptor binding, circadian entrainment, and trans-synaptic signaling pathways at 21 days post injury (Figure 4C).

### qRT-PCR and WB analysis

**Figure 5:**
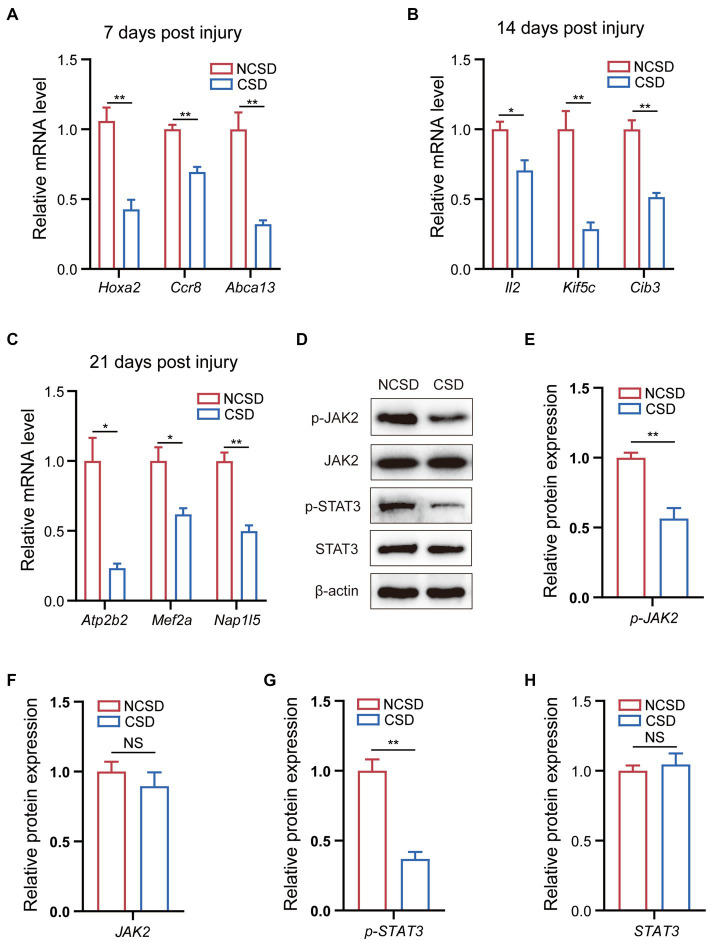
qRT-PCR and western blot results. **(A–C)** qRT-PCR analysis of the mRNA expression levels of Hox2a, Ccr8, and Abca13 at 7 days post injury, IL2, Kif5c, and Cib3 at 14 days post injury, and Atp2b2, Mef2a, and Nap1l5 at 21 days post injury. **(D)** Western blot analysis of the protein expression levels of JAK2, phospho-JAK2, STAT3, and phospho-STAT3 in bone callus from mice. **(E–H)** Relative protein expression level in the NCSD groups and CSD groups. Data are presented as means ± SEM (n = 3). *p < 0.05 and **p < 0.01, CSD, critical size defects, NCSD, non-CSD.

We established rat models of NCSD and CSD to verify the top genes at the transcriptome level and most enriched pathways at the protein level. The quantification of the mRNA expression levels of genes (Hoxa2, Ccr8, and Abca13) were reduced at 7 days post injury (Figure 5A), genes (Il2, Kif5c, and Cib3) were reduced at 14 days post injury (Figure 5B), and genes (Atp2b2, Mef2a, and Nap1l5) were reduced at 21 days post injury (Figure 5C). To verify the most enriched pathways, we detected the protein expression levels of JAK2, phospho-JAK2, STAT3, and phospho-STAT3 in callus of two groups at 14 days post injury (Figure 5D). As expected, compared with the NCSD groups, phosphorylation levels of JAK2 and STAT3 were significantly decreased in the CSD groups (Figures 5E–H).

## Discussion

Currently, it remains a challenge to repair a variety of bone defects caused by various reasons. Therefore, it is essential to understand the molecular process of bone defect progression (18). Because experimental calvarial defects in rats allow for the consistent evaluation of bone regeneration, this model has been widely accepted for the study of bone defect repair (19). It is worth mentioning that the calvarial bone formation proceeds via intramembranous ossification without intermediate cartilage formation (20, 21). GSE20980 consists of abundant transcriptome data from the regeneration region of CSD and NCSD group at 7, 14, and 21 days post injury. DEGs were identified by analyzing gene expression at these different time points, and KEGG pathway and GO enrichment analyses were subsequently conducted. PPI network was constructed to further analyze the molecular mechanism behind the progression of bone defects. In addition, qRT-PCR and WB were used to validated partial key results. This study may provide a basis for our further understanding of this clinical condition.

In this present study, there were 2,057, 827, and 1,024 DEGs at 7, 14, and 21 days post injury, respectively, at the site of new bone formation. These results indicated that we could focus on different changes in molecular events at these three time points. The majority of alterations in molecular events occurred at 7 days post injury. There were 1,754 downregulated genes at this time point. The KEGG analysis, GO enrichment analysis, and PPI analyses consistently showed that the downregulated DEGs were primarily associated with metabolic pathways and cellular metabolic process at 7 days after the bone injury. Previous research has found that inflammation plays an important role in fracture healing, and that interfering with any inflammation-related pathways or proteins will either promote or inhibit fracture healing (22, 23). A previous study has found that at 7 days after injury, macrophages undergoing metabolic reprogramming toward aerobic glycolysis inhibits fracture healing by influencing inflammation (24). Mesenchymal stem cell metabolism and osteoblast metabolism also have important roles in the fracture repair process (25). Our results are therefore a reminder to prioritize consider the metabolic pathways and cellular metabolic process in the first week post injury.

In this study, we found that the G protein-coupled signaling pathway and JAK–STAT signaling pathway played key roles at 14 days after the bone injury. The largest family of cell surface molecules involved in signaling consists of G-protein coupled receptors (GPCRs), which play key physiological roles, and their dysfunction leads to several diseases, such as osteoporosis, osteoarthritis, and ischemic stroke seizures (26, 27). GPCRs are membrane receptors that can trigger intracellular signals (28, 29). Many GPCRs [e.g., parathyroid hormone 1 receptor (PTH1R) and calcium-sensing receptor (CaSR)] can promote osteoblast differentiation, and osteoclasts also express many GPCRs [e.g., calcitonin receptor (CTR) was able to inhibit bone formation in normal bone metabolism, and ovarian cancer G-protein coupled receptor 1 (ORG1) has also been found to be promoted on osteoclast differentiation] (30–33). Together, they affect bone formation and development. In mammals, the JAK–STAT signaling pathway is critical for many cytokines and growth factors. This signaling pathway plays an important role in bone development and homeostasis, especially the STAT3, and osteoblasts and osteoclasts also express multiple JAKs and STATs (34, 35). Genetic variations in STAT3 can decrease bone mass and raise the possibility of trauma fractures in humans (34). Previous research has shown that interleukin-6 (IL-6) can promote osteoblast differentiation by activating the JAK–STAT signaling pathway (36). Thus, research focus on the processes of GPCRs and the JAK–STAT signaling pathway at 14 days post injury may contribute to the development of new bone defect treatment methods. In our study, the protein expression of phospho-JAK2 and phospho-STAT3 were found to be downregulated in CSD group at 14 days post injury. These findings may provide a potentially effective treatment strategy for orthopedic related diseases.

Notably, numerous downregulated DEGs were enriched in neurological and synaptic-related functions at 21 days post injury. Previous studies have shown that neurological damage (e.g., traumatic brain injury and spinal cord injury) can accelerate fracture healing (37, 38), and the nervous system can regulate bone homeostasis (23, 39). However, few studies have focused on synaptic alteration in bone repair regulation and the underlying mechanisms remain unclear. Such synaptic alterations warrant further investigation.

In recent years, a growing number of studies have focused on the effects of circadian rhythms on bone homeostasis (40–43). Coincidentally, in our study, we found that the regulation of the circadian rhythm may be related to bone healing at 21 days post injury. Previous studies have shown that an impaired circadian rhythm will lead to skeletal health disorders (40). For example, knocking out clock genes in bone cells can influence bone homeostasis (44, 45). Therefore, circadian entrainment should be further investigated as a potential research direction for bone healing purposes.

A key limitation of our study was that we had no raw data at 4 weeks, 5 weeks, or more subsequent time points post injury, limiting the information obtained regarding the molecular progress. Furthermore, there is still controversy over the definition of CSD, as the classical theory holds that this defect cannot be completely healed throughout the animal’s natural life. Another definition is that this time period is the entire experimental process. This involves the choice of the diameter of the defect. Additionally, we had only performed a vertical comparison by time-point grouping, and we did not find some transcriptomes that have an impact throughout each time point.

Notwithstanding, the present study revealed that three sets of molecular processes [i.e., the metabolic pathways (at 7 days post injury); the G-protein coupled signaling pathway and JAK–STAT signaling pathway (at 14 days post injury); and circadian entrainment and synaptic-related functions (at 21 days post injury)] served important roles in the progression of bone defects. These related genes may provide new insights into the treatment of orthopedic diseases, like bone defects, non-union, and fractures, or even to address systemic conditions, such as skeletal disorders and osteoporosis. However, the roles of these molecular processes in the progression of bone defects still need to be confirmed by further studies.

## Data availability statement

The datasets presented in this study can be found in online repositories. The names of the repository/repositories and accession number(s) can be found below: https://www.ncbi.nlm.nih.gov/geo/, GSE20980.

## Author contributions

SZ and GY designed the present study. HL, YL, XZ, and JY performed the experiments, data analysis, statistical analysis, and wrote the manuscript. HL, YL, XZ, CZ, ZZ, and SD were involved in the revision of the manuscript. All authors contributed to the article and approved the submitted version.

## Funding

This study was supported by grants from the National Natural Science Foundation of China (82030069, 81772351, 8151001184, and 82102570).

## Conflict of interest

The authors declare that the research was conducted in the absence of any commercial or financial relationships that could be construed as a potential conflict of interest.

## Publisher’s note

All claims expressed in this article are solely those of the authors and do not necessarily represent those of their affiliated organizations, or those of the publisher, the editors and the reviewers. Any product that may be evaluated in this article, or claim that may be made by its manufacturer, is not guaranteed or endorsed by the publisher.

## Footnotes

^1^ http://www.ncbi.nlm.nih.gov/geo
